# QuEChERS-高效液相色谱-串联质谱法同时测定豆芽中40种植物生长调节剂、杀菌剂、杀虫剂和抗生素类药物残留

**DOI:** 10.3724/SP.J.1123.2021.12028

**Published:** 2022-09-08

**Authors:** Junjun FENG, Haiyun JIANG, Jing WANG, Zhengyi JING, Fan ZHANG, Tianyu TAN, Feng HE, Lihua JIANG, Haiqin LI, Shimin CHANG, Tengfei LI

**Affiliations:** 1.河北工程大学生命科学与食品工程学院, 河北 邯郸 056038; 1. College of Life Science and Food Engineering, Hebei University of Engineering, Handan 056038, China; 2.邯郸市食品药品检验中心, 河北 邯郸 056004; 2. Food and Drug Inspection Center of Handan City, Handan 056004, China; 3.河北工程大学材料科学与工程学院, 河北 邯郸 056038; 3. College of Materials Science and Engineering, Hebei University of Engineering, Handan 056038, China

**Keywords:** QuEChERS, 高效液相色谱-串联质谱, 植物生长调节剂, 杀菌剂, 杀虫剂, 抗生素, 豆芽, QuEChERS, high performance liquid chromatography-tandem mass spectrometry (HPLC-MS/MS), plant growth regulators, fungicides, insecticide, antibiotics, bean sprout

## Abstract

研究从豆芽质量安全监管的实际需求出发,建立了QuEChERS-高效液相色谱-串联质谱同时测定豆芽中植物生长调节剂、杀菌剂、杀虫剂和抗生素类等40种药物残留的方法。首先通过参数优化确定最佳质谱条件,然后比较不同提取溶剂(甲醇、乙腈、0.1%氨水乙腈、1%乙酸乙腈)、提取方法(超声、振荡)以及乙二胺-*N*-丙基硅烷化硅胶(PSA)、C_18_净化剂添加量时40种药物的回收率,确定最优前处理过程。样品用10 mL 1%乙酸乙腈提取两次,超声波辅助提取,100 mg C_18_为净化剂。选用Waters ACQUITY UPLC BEH C_18_色谱柱(100 mm×2.1 mm, 1.7 μm)进行分离,甲醇和0.01%甲酸水为流动相梯度洗脱,多反应监测(MRM)模式进行质谱监测,基质匹配外标法定量。结果表明,40种药物可在15 min内完成色谱分离,在2~200 μg/L的线性范围内均呈现出良好的线性关系,相关系数(*r*^2^)均大于0.99,检出限(LOD)和定量限(LOQ)分别为0.1~3 μg/kg和0.3~9 μg/kg。以阴性豆芽为基质,分别在5、10、50 μg/kg 3个水平下进行加标回收试验,40种药物的平均回收率为78.5%~115.3%,相对标准偏差(RSD)为1.3%~9.7%(*n*=6)。将该方法用于分析邯郸本地豆芽中40种药物的污染状况,结果显示4-氯苯氧乙酸、6-苄基腺嘌呤、多菌灵、2,4-二氯苯氧乙酸(2,4-D)、赤霉素和恩诺沙星检出率较高,分别为28.6%、19.0%、9.5%、9.5%、4.8%和4.8%,含量范围为37.5~352.4、32.4~273.1、28.8~38.7、16.1~20.2、19.9和13.6 μg/kg。研究建立的方法简单、快速,灵敏度高,适用于大批量豆芽中40种药物的快速准确测定。

## Abstract (en)

Chromatography combined with mass spectrometry is the most commonly used detection technology, and it offers the advantages of high sensitivity and high selectivity. The quick, easy, inexpensive, effective, rugged, and safe (QuEChERS) method is low-cost, effective, and time efficient. The application of the QuEChERS has now been extended to the analysis of contaminants in food samples. The aim of the study was to identify different concentration levels of multiple harmful drug residues in bean sprouts. In this study, QuEChERS coupled with high performance liquid chromatography-tandem mass spectrometry (HPLC-MS/MS) was established for the simultaneous determination of 40 plant growth regulators, fungicides, insecticides, and antibiotics in bean sprouts. In the HPLC-MS/MS experiment, gibberellic acid, 4-fluorophenoxyacetic acid, chloramphenicol, *N*6-(*δ*2-isopentenyl)-adenine, 6-benzylaminopurine, 4-chlorophenoxyacetic acid, and 2,4-dichlorophenoxyacetic acid (2,4-D) were analyzed by MS/MS with negative electrospray ionization (ESI^-^). The other 33 target analytes (chlormequat, ronidazole, metronidazole, pymetrozine, dimetridazole, methomyl, carbendazim, enoxacin, levofloxacin, pefloxacin mesylate, norfloxacin, ciprofloxacin, enrofloxacin, thiabendazole, lomefloxacin, chlorpyrifos, sarafloxacin, imidacloprid, etc.) were analyzed by MS/MS with positive electrospray ionization (ESI^+^). Sensitive MS conditions were realized by optimizing the instrumental parameters such as the desolvent temperature, collision energy, spraying needle position, precursor ions, and product ions. Then, the optimal pretreatment method was determined by comparing the recovery rates of the 40 drugs obtained with different extraction solvents (methanol, acetonitrile, acetonitrile containing 0.1% ammonia, acetonitrile with 1% acetic acid), different extraction methods (ultrasonic extraction, shaking extraction), and purification with primary secondary amine (PSA) and C_18_. In this study, the bean sprouts samples were extracted twice by 10 mL acetonitrile with 1% acetic acid, and extracted under ultrasonic conditions. Then, the extracting solution was only cleaned with 100 mg C_18_. The chromatographic separation of the 40 compounds was accomplished on a Waters ACQUITY UPLC BEH C_18_ column (100 mm×2.1 mm, 1.7 μm) with gradient elution. Methanol and 0.01% formic acid aqueous solution were used as the mobile phases. The 40 compounds were analyzed in the multiple reaction monitoring (MRM) mode. The matrix matching external standard method was used for quantitative determination. The results showed that the 40 compounds could be analyzed within 15 min. Under the optimized conditions, the calibration curves showed good linearities for the 40 compounds, and the coefficients of determination (*r*^2^) were greater than 0.99 in the range of 2-200 μg/L. The limits of detection (LODs) and limits of quantification (LOQs) were in the range of 0.1-3 μg/kg and 0.3-9 μg/kg, respectively. Using negative bean sprouts as the substrates, the recovery tests were carried out at three spiked levels of 5, 10, and 50 μg/kg. The average recoveries of the 40 drugs were 78.5% to 115.3%, and the corresponding relative standard deviations (RSDs) were 1.3% to 9.7% (*n*=6). This method was successfully applied to the analysis of the 40 drug residues in 21 batches of local bean sprouts in Handan city. The results revealed the presence of extensive drug residues in the bean sprouts. The 26 batches were detected to varying degrees, among which 4-chlorophenoxyacetic acid, carbendazim, 6-benzyladenine, 2,4-D, enrofloxacin, and metronidazole were detected at high rates. The detection rates of 4-chlorophenoxyacetic acid, 6-benzyladenine, carbendazim, 2,4-D, gibberellic acid, and enrofloxacin were 28.6%, 19.0%, 9.5%, 9.5%, 4.8%, and 4.8%, respectively. The contents ranged from 37.5-352.4, 32.4-273.1, 28.8-38.7, 316.1-20.2, 19.9 and 13.6 μg/kg, respectively. Given its advantages of simplicity, rapidness, and high sensitivity, the developed method can be used for the rapid and accurate determination of trace levels of the 40 drug residues in large quantities of bean sprouts.

豆芽又叫“芽苗菜”或“活体蔬菜”,具有较高的营养价值,富含多种维生素、矿物质、游离氨基酸等对人体健康有益的成分^[1⇓-3]^。近年来“毒豆芽”事件频发,豆芽中检出植物生长调节剂、杀菌剂以及抗生素的事件时有报道^[4,5]^,严重威胁消费者健康。植物生长调节剂可促进植物芽的形成,抑制植物根的生长。杀菌剂、杀虫剂可杀灭豆芽中的细菌、霉菌及害虫。抗生素类药物可防止根部腐烂,从而起到保鲜的作用。长期或大量食用这类“毒豆芽”会引起肝损害、过敏反应或诱导病原体产生耐药性。

目前,豆芽中植物生长调节剂和抗生素检测方法均有相关标准,国家食品监督管理局最新发布的BJS 201703《豆芽中植物生长调节剂的测定》可测定豆芽中11种植物生长调节剂^[6]^;国家市场监督管理总局发布的BJS 201909《豆制品、火锅、麻辣烫等食品中喹诺酮类化合物的测定》可测定豆制品、火锅、麻辣烫等食品中的11种喹诺酮类化合物^[7]^; 《豆芽中氟喹诺酮类和硝基咪唑类药物残留量的测定 液相色谱-串联质谱法》(T/ZACA 021-2020)可同时测定12种抗生素^[8]^。而能够同时测定豆芽中多种不同类别的植物生长调节剂、杀菌剂、杀虫剂以及抗生素的研究还未见报道。当前的检测方法大多目标化合物类别单一,且前处理繁琐、耗时,不能满足快速、高效、准确,以及多目标物等处置食品安全突发事件的要求,因此选择快速、高效的前处理方法并建立同时测定多种药物残留的分析方法对保障豆芽质量安全具有重要意义。

QuEChERS法具有快速、简单、廉价、高效、安全等特点,广泛应用于农产品检测的前处理过程中。本研究依据相关文献^[9⇓⇓⇓⇓⇓⇓⇓⇓⇓⇓⇓⇓⇓⇓⇓⇓⇓⇓-28]^及豆芽质量安全监管需求,选择了植物生长调节剂、杀菌剂、杀虫剂以及抗生素4种药物作为目标化合物,应用改进的QuEChERS法,结合高效液相色谱-串联质谱技术,建立了同时测定豆芽中40种药物残留的方法,并对21批豆芽中的药物残留进行了分析。该方法拓展了现有检测方法中涵盖目标物的种类,缩短了检测时间,为加强豆芽的质量安全监管提供技术支撑。

## 1 实验部分

### 1.1 仪器、试剂与材料

LCMS-8030液相色谱-三重四极杆质谱仪、AUW120D电子天平、LabSolutions工作站(日本Shimadzu公司); KQ-250DE型数控超声波清洗器(昆山市超声仪器有限公司); Direct-Q超纯水仪(美国Millipore公司); Heraeus^TM^ Megafuge^TM^ 8R冷冻离心机(美国Thermo公司); Vortex-Genie 2涡旋振荡器(美国Scientific Industries公司); N-EVAP-112氮吹仪(美国Organomation公司)。

甲醇、乙腈、乙酸(色谱纯)购自美国Thermo公司;甲酸为色谱纯,购自北京百灵威科技有限公司;C_18_(50 μm)购自天津博纳艾杰尔科技有限公司;氯化钠(分析纯)购自天津市瑞金特化学品有限公司;无水硫酸镁(分析纯)购自天津欧博凯化工有限公司;实验用水为超纯水;实际样品购自本地超市和市场。

11种植物生长调节剂混合标准溶液(赤霉素、4-氟苯氧乙酸、吲哚乙酸、异戊烯腺嘌呤、6-苄基腺嘌呤、4-氯苯氧乙酸、噻苯隆、吲哚丁酸、氯吡脲、2,4-二氯苯氧乙酸(2,4-D)、多效唑)、7种喹诺酮类药物混合标准溶液(氧氟沙星、培氟沙星、诺氟沙星、环丙沙星、恩诺沙星、洛美沙星、沙拉沙星)、依诺沙星、司帕沙星、矮壮素、莠去津、氟硅唑等标准溶液(纯度均>98.5%)购自美国A Chemtek公司;洛硝哒唑、甲硝唑、吡蚜酮、二甲硝咪唑、灭多威、多菌灵、毒死蜱、吡唑醚菌酯、氯霉素标准溶液(纯度均>98%)购自迈迪嘉(天津)科技有限公司;啶虫脒、噻菌灵、吡虫啉、精甲霜灵、甲霜灵、嘧菌酯、三唑磷、氯唑磷等标准溶液(纯度均>99%)购自农业农村部环境保护科研监测所。

### 1.2 标准溶液的制备

混合标准储备液:分别移取40种标准溶液适量,以甲醇为溶剂配成质量浓度为10 mg/L的混合标准储备液,于-18 ℃冰箱储存备用。

混合标准中间溶液:分别吸取10 mg/L的混合标准储备液1 mL于10 mL容量瓶中,用甲醇定容,配制成质量浓度为1 mg/L的混合标准溶液,密封储存于-18 ℃冰箱。

基质匹配混合标准溶液:分别向1 mL容量瓶中加入2、5、10、20、50、100、200 μL 1 mg/L的混合标准储备液,用空白样品提取液定容至刻度,配制成2、5、10、20、50、100、200 μg/L的基质匹配混合标准溶液,现配现用。

### 1.3 样品前处理

准确称取粉碎均匀的豆芽样品5.00 g于50 mL聚丙烯离心管中,加入1颗陶瓷均质子、4 g无水硫酸镁和1 g NaCl后立即涡旋混合1 min,加入10 mL 1%(v/v,下同)乙酸乙腈溶液,涡旋混合1 min,超声提取15 min, 8000 r/min冷冻离心5 min,将上清液倾入50 mL具塞离心管中,残渣再用10 mL 1%乙酸乙腈溶液重复提取1次,合并上清液。移取10 mL上清液在40 ℃氮吹至近干,加入1 mL 5%甲醇水溶液溶解残渣,涡旋均匀后加入称有100 mg C_18_的10 mL塑料离心管中,涡旋混合2 min, 5000 r/min离心5 min,过0.22 μm有机滤膜后上机测试。

### 1.4 色谱条件

色谱柱:Waters ACQUITY UPLC BEH C_18_柱(100 mm×2.1 mm, 1.7 μm);柱温:40 ℃;流速:0.3 mL/min;二元流动相:A为0.01%甲酸水溶液,B为甲醇。梯度洗脱程序:0~3 min, 5%B; 3~7 min, 5%B~30%B; 7~9 min, 30%B~85%B; 9~13 min, 85%B; 13~13.01 min, 85%B~5%B; 13.01~15 min, 5%B。进样体积:10 μL。

### 1.5 质谱条件

电喷雾电离(ESI)源,正离子模式和负离子模式扫描;接口电压:4.5 kV;脱溶剂气温度:250 ℃;加热块温度:450 ℃;干燥气流速:15 L/min;雾化气流速:3 L/min;多反应监测(MRM)模式。40种化合物的保留时间和质谱参数见表1。

**表 1:** MRM模式下40种化合物的保留时间与质谱参数

No.	Compound	Retention time/min	Ionization mode	Precursor ion (m/z)	Product ions (m/z)	Collision energies/eV
1	chlormequat (矮壮素)	1.066	ESI^+^	122.10	58.10^*^, 63.10	-31, -24
2	ronidazole (洛硝哒唑)	3.903	ESI^+^	201.00	139.70^*^, 54.60	-10, -15
3	metronidazole (甲硝唑)	3.999	ESI^+^	172.00	81.80^*^, 127.70	-25, -15
4	pymetrozine (吡蚜酮)	4.192	ESI^+^	408.10	105.05^*^, 68.10	-39, -30
5	dimetridazole (二甲硝咪唑)	5.211	ESI^+^	142.10	96.05^*^, 81.10	-16, -27
6	methomyl (灭多威)	6.850	ESI^+^	163.05	88.00^*^, 106.05	-11, -13
7	carbendazim (多菌灵)	6.959	ESI^+^	192.05	160.05^*^, 32.05	-17, -30
8	levofloxacin (氧氟沙星)	7.261	ESI^+^	362.10	318.20^*^, 61.10	-20, -30
9	enoxacin (依诺沙星)	7.272	ESI^+^	340.20	303.10^*^, 203.85	-20, -48
10	pefloxacin mesylate (培氟沙星)	7.301	ESI^+^	334.00	290.10^*^, 16.10	-20, -20
11	norfloxacin (诺氟沙星)	7.384	ESI^+^	320.00	302.00^*^, 76.00	-20, -17
12	ciprofloxacin (环丙沙星)	7.537	ESI^+^	332.20	314.05^*^, 31.00	-24, -41
13	enrofloxacin (恩诺沙星)	7.672	ESI^+^	360.20	316.10^*^, 42.15	-22, -25
14	thiabendazole (噻菌灵)	7.749	ESI^+^	202.00	175.05^*^, 31.10	-24, -33
15	lomefloxacin (洛美沙星)	7.749	ESI^+^	352.00	265.00^*^, 80.10	-25, -20
16	chlorpyrifos (毒死蜱)	7.768	ESI^+^	351.90	265.05^*^, 37.05	-30, -39
17	sarafloxacin (沙拉沙星)	8.048	ESI^+^	385.90	367.95^*^, 98.90	-25, -20
18	imidacloprid (吡虫啉)	8.172	ESI^+^	256.05	175.10^*^, 09.05	-17, -14
19	sparfloxacin (司帕沙星)	8.480	ESI^+^	393.00	349.00^*^, 92.00	-40, -20
20	acetamiprid (啶虫脒)	8.682	ESI^+^	223.10	126.05^*^, 56.05	-22, -15
21	gibberellic acid (赤霉素)	8.744	ESI^-^	345.40	143.20^*^, 39.50	32, 16
22	4-fluorophenoxyacetic acid (4-氟苯氧乙酸)	8.941	ESI^-^	169.20	111.00^*^, 95.00	16, 18
23	chloramphenicol (氯霉素)	9.144	ESI^-^	340.00	152.10^*^, 57.10	17, 11
24	3-indolylacetic acid (吲哚乙酸)	9.230	ESI^+^	176.10	129.80^*^, 03.10	-18, -16
25	N6-(δ2-isopentenyl)-adenine (异戊烯腺嘌呤)	9.434	ESI^-^	202.30	133.00^*^, 34.15	22, 17
26	6-benzylaminopurine (6-苄基腺嘌呤)	9.487	ESI^-^	224.00	133.00^*^, 06.00	40, 34
27	4-chlorophenoxyacetic acid (4-氯苯氧乙酸)	10.040	ESI^-^	185.00	127.10^*^, 29.00	15, 14
28	thidiazuron (噻苯隆)	10.056	ESI^+^	240.20	102.00^*^, 28.00	-16, -17
29	3-indolebutyric acid (吲哚丁酸)	10.229	ESI^+^	204.20	186.20^*^, 29.90	-10, -28
30	atrazine (莠去津)	10.556	ESI^+^	406.10	174.05^*^, 96.05	-17, -25
31	forchlorfenuron (氯吡脲)	10.595	ESI^+^	248.05	129.05^*^, 93.05	-17, -34
32	metalaxyl-M (精甲霜灵)	10.614	ESI^+^	280.10	220.15^*^, 48.10	-13, -10
33	metalaxyl (甲霜灵)	10.619	ESI^+^	280.10	220.15^*^, 92.15	-13, -18
34	2,4-dichlorophenoxyacetic acid (2,4-滴)	10.669	ESI^-^	409.00	160.85^*^, 25.00	14, 29
35	azoxystrobin (嘧菌酯)	10.826	ESI^+^	404.10	372.05^*^, 29.00	-14, -31
36	paclobutrazol (多效唑)	11.064	ESI^+^	294.10	70.05^*^, 125.05	-40, -40
37	triazophos (三唑磷)	11.152	ESI^+^	314.05	162.15^*^, 19.15	-19, -35
38	isazophos (氯唑磷)	11.161	ESI^+^	314.10	162.10^*^, 20.10	-16, -27
39	flusilazole (氟硅唑)	11.394	ESI^+^	316.10	247.10^*^, 65.10	-18, -29
40	pyraclostrobin (吡唑醚菌酯)	11.635	ESI^+^	388.10	194.05^*^, 63.05	-30, -32

* Quantitative ion.

## 2 结果与讨论

### 2.1 质谱条件的优化

在多反应监测、正、负离子模式下,分别对脱溶剂温度、碰撞能量、喷针位置和选择离子等进行了充分优化,选取经碰撞后所得丰度较高的两个子离子作为定量和定性离子,并确定最佳碰撞能量。最终所选择确定的40种化合物的电离方式、母离子、子离子、碰撞能量及保留时间等参数见表1。

### 2.2 色谱条件的优化

实验选用Waters ACQUITY UPLC BEH C_18_色谱柱(100 mm×2.1 mm, 1.7 μm)分离40种目标化合物。实验结果表明:采用此色谱柱时,40种化合物均可得到有效分离,且色谱峰峰形较好。

在流动相的选择实验中,首先考察甲醇和乙腈。经过比较,当采用乙腈为流动相时,大多数化合物分离效果优于甲醇,灵敏度更高,但甲硝唑、喹诺酮类等部分化合物出峰时间过早,且出现双峰;而采用甲醇为流动相时,各物质均可获得较好的分离度及较高的响应值,故选择甲醇为强洗脱有机相。

实验比较了水、0.01%甲酸水溶液、0.02%甲酸水溶液、0.03%甲酸水溶液、0.1%甲酸水溶液、2 mmol/L乙酸铵水溶液(含0.02%甲酸)等6种水相对40种化合物灵敏度及分离度的影响。实验发现:当使用纯水为水相时,赤霉素峰形分裂,喹诺酮类化合物响应较低,且大部分出现分叉、拖尾现象;加入0.01%甲酸后,吡蚜酮及喹诺酮类化合物峰形尖锐,灵敏度提高,但降低了赤霉素、氯霉素、2,4-D等部分化合物的峰响应值。这是由于加入甲酸可以提高吡蚜酮等化合物的离子化效率,促进了[M+H]^+^峰的形成,提高了质谱响应且改善了峰形。但加入甲酸降低了赤霉素、氯霉素、2,4-D等化合物的离子化效率,抑制了[M-H]^-^峰的形成,降低了其在质谱上的响应值。进一步增大甲酸浓度,毒死蜱、矮壮素等化合物色谱峰响应值变化不大,但大大降低了氯霉素、6-苄基腺嘌呤等化合物色谱峰的响应值。当使用2 mmol/L乙酸铵水溶液(含0.02%甲酸)为水相时,虽可提高氯霉素、6-苄基腺嘌呤等部分化合物响应值,但会使得喹诺酮类化合物峰形变差;故经反复试验后选择0.01%甲酸水溶液为水相。

在该流动相体系下,40种化合物可达到较好分离。40种化合物的标准溶液(50 μg/L)总离子流(TIC)色谱图见图1。

**图 1:**
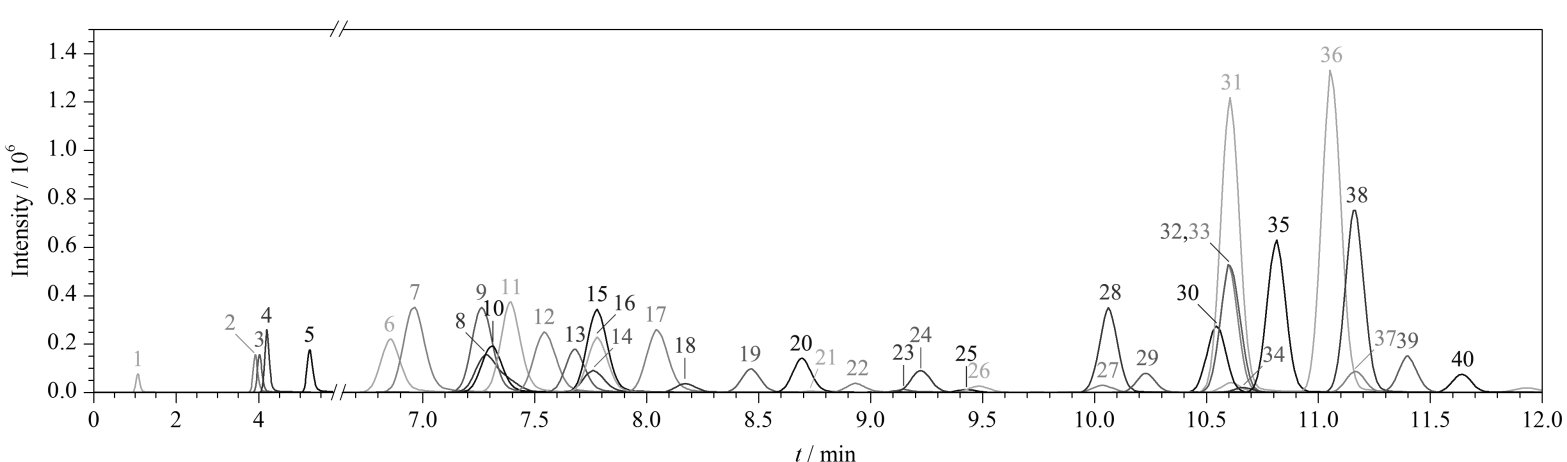
40种化合物标准溶液的总离子流图

### 2.3 提取条件的优化

#### 2.3.1 提取溶剂的选择

为了更好地提取豆芽中40种化合物,通过添加回收的方式分别比较了甲醇、乙腈、1%氨水乙腈、1%乙酸乙腈作为提取溶剂时的提取效果,添加水平为20 μg/kg。

实验结果表明:采用甲醇、乙腈、1%氨水乙腈时的回收率明显低于乙腈,采用1%乙酸乙腈时的回收率最高,均高于78%。这是由于乙腈极性范围宽、组织穿透能力强,对大多数目标化合物有较好的溶解性和提取效率,且对豆芽基质中的脂肪、色素等有一定的去除能力,并减少基质对目标化合物的干扰。加入乙酸不仅可以破坏豆芽基质的组织细胞,而且可以抑制赤霉素、吲哚乙酸、2,4-D等目标化合物中羧基的解离,从而提高提取效率。因此最终选用1%乙酸乙腈作为提取溶剂。

#### 2.3.2 提取方式的选择

本实验比较了超声、振荡两种提取方式,通过回收率来验证40种化合物的提取效果。添加水平为20 μg/kg,每种提取方式做3个平行。实验结果表明:在最优条件下,采取超声提取的方式优于振荡提取,40种化合物的平均回收率为79%~111%。这可能是因为豆芽在生长过程中添加的药物会通过代谢活动进入到组织细胞中,且还可能会以其他化合物形态存在,目标化合物与组织细胞结合较为紧密,单纯的振荡形式无法有效提取目标化合物。因此,本实验选用超声作为提取方式。

### 2.4 净化剂用量的优化

QuEChERS净化法常用的吸附净化剂有PSA、C_18_、石墨化炭黑(GCB),其中PSA可以吸附基质中的糖类、色素以及脂肪酸^[29]^。C_18_可以吸附脂肪和一些矿物质。GCB能去除部分色素、固醇和具有平面结构等极性大的基质的干扰^[30]^,但对仪器检测器识别起干扰作用,且对含有平面芳香环结构的农药分子有较强的吸附作用,如多菌灵^[31]^,考虑到豆芽中色素的干扰不明显,故不使用GCB作为净化吸附剂。在实验过程中,发现加入PSA使得极性较强的矮状素以及吲哚乙酸、吲哚丁酸、赤霉素等含羧基化合物的回收率降低,其原因可能是由于PSA为碱性,对极性较强的矮状素以及含有羧酸类的吲哚乙酸、吲哚丁酸等化合物有吸附作用。因此本实验比较了不同用量的C_18_吸附剂对目标化合物回收率的影响,见图2。结果表明:C_18_添加量在20~120 mg范围内,各化合物的回收率差别不大。因此综合考虑回收率及除杂效果,本实验选择100 mg C_18_作为净化吸附剂。

**图 2:**
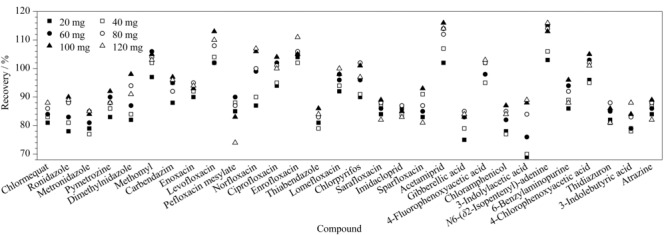
不同用量的C_18_对目标化合物回收率的影响

### 2.5 基质效应评价

为了消除样品基质对目标化合物测定结果的影响,本实验采用提取后添加法^[32]^,将阴性黄豆芽和绿豆芽按1.3节前处理方法处理后,配制50 μg/L的基质混合标准溶液,同时采用纯溶剂配制相同浓度的混合标准溶液,按式(1)通过计算两者对应的目标化合物响应值的比值作为基质效应评估值。


(1) $$ME=\frac{A_{2}}{A_{1}}$$

式中,ME表示基质效应大小。当ME<1.0,则基质对待测物的响应产生抑制作用;当ME>1.0,则基质对待测物的响应产生增强作用;当ME=1.0,则说明基质对待测物没有影响,这是一种理想状态。*A*_1_为纯溶剂中化合物的响应值;*A*_2_为空白基质溶液中该化合物的响应值。

结果显示:黄豆芽中40种化合物基质效应为0.74~2.21,绿豆芽中40种化合物基质效应为0.61~2.15,均存在一定的抑制或增强效应。故为降低基质效应的影响,本实验采用空白基质提取液配制混合标准溶液进行校正,经校正后可有效消除基质效应带来的影响,提高检测结果的准确性。

### 2.6 方法学验证

#### 2.6.1 线性范围、检出限和定量限

按仪器工作条件对40种目标化合物的基质匹配混合标准溶液进行测定,以40种目标化合物的质量浓度为横坐标,以对应的峰面积为纵坐标绘制标准曲线,并求出相应的线性回归方程及相关系数(*r*^2^)。在空白样品中添加一定浓度的40种目标化合物的混合标准溶液,按1.3节进行前处理后上机测定。以3倍信噪比确定方法的检出限(LOD),以10倍信噪比确定方法的定量限(LOQ)。40种目标化合物的线性范围、线性方程、相关系数、检出限和定量限见表2。结果表明:40种目标化合物在2~200 μg/L的范围内与其峰面积呈良好的线性关系,*r*^2^均达0.99以上,检出限为0.1~3.0 μg/kg,定量限为0.3~9.0 μg/kg。

**表 2:** 40种化合物的线性方程、线性范围、相关系数、检出限和定量限

Compound	Linear equation	Linear range/(μg/L)	r^2^	LOD/(μg/kg)	LOQ/(μg/kg)
Chlormequat	y=1.27×10^3^x-5.36×10^2^	2-200	0.9966	1.5	4.5
Ronidazole	y=1.82×10^4^x-9.28×10^3^	2-200	0.9987	0.1	0.3
Metronidazole	y=1.28×10^4^x+4.55×10^3^	2-200	0.9998	0.5	1.5
Pymetrozine	y=2.98×10^4^x-1.13×10^5^	2-200	0.9929	0.5	1.5
Dimetridazole	y=3.14×10^4^x-1.22×10^4^	2-200	0.9997	0.2	0.6
Methomyl	y=1.43×10^4^x-3.36×10^4^	2-200	0.9914	0.2	0.6
Carbendazim	y=5.18×10^4^x+3.92×10^4^	2-200	0.9998	0.2	0.6
Enoxacin	y=2.01×10^4^x-1.33×10^4^	2-200	0.9919	1.0	3.0
Levofloxacin	y=2.07×10^4^x-6.38×10^4^	2-200	0.9939	1.0	3.0
pefloxacin mesylate	y=1.07×10^4^x-2.77×10^4^	2-200	0.9959	1.0	3.0
Norfloxacin	y=2.86×10^4^x-1.09×10^4^	2-200	0.9977	0.5	1.5
Ciprofloxacin	y=1.01×10^4^x+1.21×10^4^	2-200	0.9939	1.0	3.0
Enrofloxacin	y=1.45×10^4^x-6.98×10^4^	2-200	0.9932	1.0	3.0
Thiabendazole	y=1.26×10^4^x-1.20×10^4^	2-200	0.9976	0.5	1.5
Lomefloxacin	y=3.72×10^4^x-1.31×10^3^	2-200	0.9917	0.5	1.5
Chlorpyrifos	y=2.17×10^4^x-4.32×10^4^	2-200	0.9903	0.5	1.5
Sarafloxacin	y=2.25×10^4^x-7.64×10^4^	2-200	0.9953	0.5	1.5
Imidacloprid	y=3.84×10^3^x-1.55×10^4^	2-200	0.9908	1.0	3.0
Sparfloxacin	y=9.99×10^3^x-4.35×10^4^	2-200	0.9906	0.5	1.5
Acetamiprid	y=1.18×10^4^x-1.79×10^4^	2-200	0.9948	0.5	1.5
Gibberellic acid	y=5.95×10^4^x-2.08×10^3^	2-200	0.9914	3.0	9.0
4-Fluorophenoxyacetic acid	y=6.08×10^3^x-3.00×10^3^	2-200	0.9989	1.5	4.5
Chloramphenicol	y=8.61×10^2^x+1.86×10^3^	2-200	0.9970	3.0	9.0
3-Indolylacetic acid	y=7.02×10^3^x+1.76×10^3^	2-200	0.9961	2.0	6.0
N6-(δ2-Isopentenyl)-adenine	y=3.27×10^2^x-3.65×10^2^	2-200	0.9967	3.0	9.0
6-Benzylaminopurine	y=3.97×10^2^x-2.74×10^3^	2-200	0.9945	3.0	9.0
4-Chlorophenoxyacetic acid	y=2.78×10^3^x-2.33×10^3^	2-200	0.9995	3.0	9.0
Thidiazuron	y=5.69×10^4^x-2.10×10^4^	2-200	0.9996	0.5	1.5
3-Indolebutyric acid	y=4.55×10^3^x-4.29×10^3^	2-200	0.9967	2.0	6.0
Atrazine	y=2.23×10^4^x-7.41×10^4^	2-200	0.9956	0.2	0.6
Compound	Linear equation	Linear range/(μg/L)	r^2^	LOD/(μg/kg)	LOQ/(μg/kg)
Forchlorfenuron	y=1.83×10^5^x-3.65×10^5^	2-200	0.9977	0.1	0.3
Metalaxyl-M	y=4.74×10^4^x+1.29×10^5^	2-200	0.9981	0.2	0.6
Metalaxyl	y=2.84×10^4^x+9.92×10^4^	2-200	0.9966	0.2	0.6
2,4-Dichlorophenoxyacetic acid	y=8.84×10^3^x-3.59×10^3^	2-200	0.9995	2.0	6.0
Azoxystrobin	y=5.32×10^4^x+1.19×10^4^	2-200	0.9992	0.2	0.6
Paclobutrazol	y=2.02×10^5^x-3.79×10^2^	2-200	0.9986	0.1	0.3
Triazophos	y=6.95×10^4^x+1.02×10^5^	2-200	0.9983	0.1	0.3
Isazophos	y=6.66×10^4^x+1.08×10^5^	2-200	0.9966	0.1	0.3
Flusilazole	y=3.28×10^4^x-2.57×10^4^	2-200	0.9994	1.0	3.0
Pyraclostrobin	y=8.67×10^4^x+5.62×10^5^	2-200	0.9979	0.5	1.5

*y*: peak area; *x*: mass concentration, μg/L.

#### 2.6.2 回收率及精密度

在空白样品中分别添加5、10、50 μg/kg 3个水平的40种混合标准溶液进行加标回收试验,每个水平平行测定6次,见表3。

**表 3:** 40种化合物的回收率和精密度(*n*=6)

Compound	5 μg/kg			10 μg/kg			50 μg/kg	
	Recovery/%	RSD/%		Recovery/%	RSD/%		Recovery/%	RSD/%
Chlormequat	83.4	6.9		86.1	5.2		93.6	2.8
Ronidazole	111.2	4.3		93.2	2.4		96.2	3.7
Metronidazole	88.2	5.3		85.7	6.5		91.0	5.7
Pymetrozine	102.4	6.9		82.1	5.1		84.5	6.5
Dimetridazole	108.4	2.1		89.6	3.9		92.6	2.2
Methomyl	82.7	8.7		89.4	4.9		85.4	5.1
Carbendazim	84.3	7.5		97.6	4.7		94.1	8.0
Enoxacin	82.6	1.8		95.6	3.0		101.7	4.5
Levofloxacin	102.1	7.6		106.5	7.1		93.0	5.4
pefloxacin mesylate	90.3	7.1		101.5	5.9		92.8	4.1
Norfloxacin	84.2	5.9		86.7	4.6		88.6	4.7
Ciprofloxacin	99.5	8.3		102.2	7.2		96.4	4.3
Enrofloxacin	104.7	5.3		100.9	4.2		101.6	3.8
Thiabendazole	85.4	9.0		82.9	6.3		82.6	4.4
Lomefloxacin	97.9	6.2		105.5	6.1		99.2	2.9
Chlorpyrifos	112.6	9.7		113.1	6.3		107.4	4.1
Sarafloxacin	108.4	4.4		105.2	3.9		102.6	3.7
Imidacloprid	80.0	7.6		84.4	6.2		85.1	5.9
Sparfloxacin	106.6	5.3		98.4	3.6		102.5	5.4
Acetamiprid	105.3	3.8		111.6	3.2		115.3	4.2
Gibberellic acid	77.4	8.2		81.6	1.7		82.1	5.7
4-Fluorophenoxyacetic acid	98.6	7.2		93.6	4.8		91.9	3.3
Chloramphenicol	78.8	4.0		85.8	5.1		85.4	5.9
3-Indolylacetic acid	89.9	3.1		86.5	3.4		97.7	6.3
N6-(δ2-Isopentenyl)-adenine	92.3	1.6		87.2	6.2		96.8	4.0
6-Benzylaminopurine	102.4	6.4		94.3	5.9		95.6	5.2
4-Chlorophenoxyacetic acid	111.3	8.9		108.4	5.3		106.8	5.7
Thidiazuron	94.7	1.9		95.5	4.6		92.7	2.4
3-Indolebutyric acid	78.5	6.2		90.3	3.6		86.4	3.5
Atrazine	94.6	3.8		89.0	6.4		86.9	4.6
Compound	5 μg/kg			10 μg/kg			50 μg/kg	
	Recovery/%	RSD/%		Recovery/%	RSD/%		Recovery/%	RSD/%
Forchlorfenuron	103.4	5.3		108.2	4.2		105.7	2.7
Metalaxyl-M	112.4	4.7		98.8	2.7		106.4	4.6
Metalaxyl	97.4	3.0		108.5	1.7		102.1	3.2
2,4-Dichlorophenoxyacetic acid	85.5	8.3		83.2	8.0		87.5	6.6
Azoxystrobin	91.6	3.4		87.3	5.7		84.2	5.9
Paclobutrazol	112.6	2.2		103.5	6.1		107.6	3.6
Triazophos	82.1	9.1		84.3	7.4		88.6	5.5
Isazophos	91.6	5.5		83.6	3.8		84.3	1.9
Flusilazole	82.1	2.1		88.3	4.4		85.7	1.3
Pyraclostrobin	116.4	5.9		107.2	4.6		108.7	4.8

结果表明:40种目标化合物回收率为78.5%~115.3%,相对标准偏差(RSD)为1.3%~9.7%。测定结果均符合GB/T 27404-2008《实验室质量控制规范食品理化检测》规定,说明本方法对豆芽中40种化合物的测定均有良好的准确度和精密度。

### 2.7 实际样品的测定

为了验证本方法的可靠性,同时了解邯郸本地豆芽中药物残留的情况,采用本方法对本地超市和市场采集的21批次豆芽样品进行测定,结果见表4。其中10批次有不同程度的检出(>LOD),其余未检出。4-氯苯氧乙酸、6-苄基腺嘌呤、多菌灵、2,4-D、赤霉素和恩诺沙星检出率较高,分别为28.6%、19.0%、9.5%、9.5%、4.8%和4.8%,含量为37.5~352.4、32.4~273.1、28.8~38.7、16.1~20.2、19.9和13.6 μg/kg。从结果可以看出,豆芽中这些药物残留的风险较高。

**表 4:** 实际样品检测结果

Compound	Contents/(μg/kg)	
	This method	Standard method
4-Chlorophenoxyacetic acid	37.5-52.4	34.2-343.5
6-Benzylaminopurine	32.4-273.1	29.3-279.4
Carbendazim	28.8-38.7	-
2,4-Dichlorophenoxyacetic acid	16.1-20.2	14.1-18.3
Gibberellic acid	19.9	22.1
Enrofloxacin	13.6	15.3

-: no standard method.

同时,将实际样品中检出的4-氯苯氧乙酸、6-苄基腺嘌呤、2,4-D、赤霉素与豆芽中植物生长调节剂的测定方法(BJS 201703)进行比较,恩诺沙星与豆制品、火锅、麻辣烫等食品中喹诺酮类化合物的测定方法(BJS 201909)进行比较(见表4)。结果表明,本方法与标准方法测得结果基本一致,相对标准偏差为1.5%~8.3%,表明本方法数据准确可靠。

### 2.8 与文献方法比较

对于恩诺沙星、氧氟沙星、诺氟沙星、多菌灵、莠去津、4-氯苯氧乙酸、6-苄基腺嘌呤以及赤霉素等大部分化合物的检出限,与文献^[9,10,12,13,20⇓-22,27,33⇓⇓⇓-37]^报道的方法相比(见表5),在回收率相当的情况下,本方法的检出限低,无需过净化柱,样品前处理步骤简单、快速、灵敏、高效,且一次可以完成40种化合物的测定,扩展了豆芽中药物检测种类,有效提高了日常监测效率。

**表 5:** 本方法与文献方法的比较

Compound	LODs/(μg/kg)	
	Reference	This work
Chlormequat	5^[33]^	0.2
Carbendazim	45^[22]^	0.2
Ronidazole	0.4^[37]^	0.1
Dimethylnidazole	0.5^[10]^	0.2
Carbendazim	3.7^[27]^	0.2
Enoxacin	2^[35]^	1.0
Levofloxacin	2^[20]^	1.0
pefloxacin mesylate	2^[35]^	1.0
Norfloxacin	64^[22]^	0.5
Ciprofloxacin	2^[35]^	1.0
Enrofloxacin	2^[20]^	1.0
Thiabendazole	2.1^[27]^	0.5
Lomefloxacin	2^[35]^	0.5
Sarafloxacin	2^[35]^	0.5
Sparfloxacin	2^[35]^	0.5
Gibberellic acid	6.0^[21]^	3.0
4-Fluorophenoxyacetic acid	10^[36]^	1.5
Chloramphenicol	10^[9]^	2.0
3-Indolylacetic acid	72^[22]^	3.0
N6-(δ2-Isopentenyl)-adenine	10^[36]^	3.0
6-Benzylaminopurine	3.8^[12]^	3.0
4-Chlorophenoxyacetic acid	5^[34]^	3.0
Thidiazuron	10^[36]^	0.2
3-Indolebutyric acid	10^[36]^	2.0
Atrazine	1^[33]^	0.2
Forchlorfenuron	0.26^[13]^	0.1
2,4-Dichlorophenoxyacetic acid	1^[33]^	2.0
Paclobutrazol	0.6^[21]^	0.1

## 3 结论

本文建立了QuEChERS结合液相色谱-串联质谱同时检测豆芽中40种药物残留的分析方法。该方法前处理简单、快速、灵敏,定量结果准确,适用于豆芽中多种药物残留的快速检测。与现行国家标准及已有文献相比,该方法能实现豆芽中不同类别的植物生长调节剂、杀菌剂、杀虫剂以及抗生素等残留的同时检测,大大缩短了检测周期,不仅能够为我国食品安全风险监测和大量样品筛查提供技术支撑,而且可以为以后相关国家标准的制定提供重要参考。
